# Effects of scutellarin on the mechanism of cardiovascular diseases: a review

**DOI:** 10.3389/fphar.2023.1329969

**Published:** 2024-01-08

**Authors:** Xinyu Zhang, Tong Yin, Yincang Wang, Jiazhe Du, Jinjin Dou, Xiwu Zhang

**Affiliations:** ^1^ Graduate School, Heilongjiang University of Chinese Medicine, Harbin, China; ^2^ First Clinical Medical School, Heilongjiang University of Chinese Medicine, Harbin, China; ^3^ Department of Cardiovascular, The First Hospital of Heilongjiang University of Chinese Medicine, Harbin, China; ^4^ Experimental Training Centre, Heilongjiang University of Chinese Medicine, Harbin, China

**Keywords:** scutellarin, signal pathway, mechanism of action, cardiovascular system, cardioprotection

## Abstract

Cardiovascular diseases represent a significant worldwide problem, jeopardizing individuals’ physical and mental wellbeing as well as their quality of life as a result of their widespread incidence and fatality. With the aging society, the occurrence of Cardiovascular diseases is progressively rising each year. However, although drugs developed for treating Cardiovascular diseases have clear targets and proven efficacy, they still carry certain toxic and side effect risks. Therefore, finding safe, effective, and practical treatment options is crucial. Scutellarin is the primary constituent of Erigeron breviscapus (Vant.) Hand-Mazz. This article aims to establish a theoretical foundation for the creation and use of secure, productive, and logical medications for Scutellarin in curing heart-related illnesses. Additionally, the examination and analysis of the signal pathway and its associated mechanisms with regard to the employment of SCU in treating heart diseases will impart innovative resolving concepts for the treatment and prevention of Cardiovascular diseases.

## 1 Introduction

Population aging is a major problem facing mankind on a global scale. It is expected that by 2030, the global population aged 65 and above will account for one-fifth of the total population. At the same time, aging is an independent risk factor for cardiovascular disease (CVD). This will lead to an exponential increase in the prevalence of CVD (Jaiswal and Libby, 2020; Rudnicka et al., 2020). CVD poses a serious threat to individuals’ physical and emotional health and quality of life because of its high morbidity and mortality. Currently, the drugs used in the prevention and treatment of CVD have clear targets and certain efficacy, but there is also the problem of a single target and certain toxic side effects (Liau et al., 2019; Lin et al., 2020). Therefore, it is crucial to investigate more potent and safer pharmaceuticals for managing CVD.

Scutellarin (SCU) is the main active substance in the flavonoids of the Erigeron flower. In the last few years, the distinct significance of SCU in CVD has garnered significant attention. Despite the multiple pharmacological effects of SCU, researchers worldwide are still exploring its mechanism of action. Therefore, the objective of this paper is to analyze the effects of SCU on signal pathways related to CVDs and drug metabolism. The goal is to provide a reference for future applications of SCU in preventing and treating CVDs.

## 2 Scutellarin

Erigeron breviscapus (Vant.) Hand-Mazz (EBHM) is a botanical herb frequently employed in traditional Chinese medicine in the Yunnan, Hunan, and Guizhou provinces of China. Based on “Yunnan Materia Medica,” EBHM can increase blood flow, eliminate stagnant blood, unblock meridians, and alleviate pain. Based on the pharmacological effects of EBHM, researchers have developed a series of drugs, such as Erigeron breviscapus injection, Erigeron Capsules, Erigeron breviscapus granules, *etc.* These medications are frequently utilized to manage cardiovascular and cerebrovascular illnesses due to their capacity to dilate blood vessels, enhance microcirculation, inhibit platelet aggregation, decrease lipid peroxides, increase fibrinolytic activity, and reduce blood viscosity (Gao et al., 2017; Ma et al., 2023).

Active component SCU is taken out of EBHM. The wide range of pharmacological properties that SCU contains, including the ability to treat diseases and have protective benefits on the body, have been shown by contemporary pharmacological investigations. Its anti-tumor, glaucoma-improving, anti-depressive, and osteoporosis-preventive qualities are a few of these functions (Lu et al., 2021; Zhu et al., 2021; Chen et al., 2022a; Teng et al., 2022). Multiple benefits of SCU on cardiovascular illnesses include anti-myocardial fibrosis, protection of vascular endothelial function, attenuation of myocardial injury, and improvement of cardiac function (Xu et al., 2020; Duan et al., 2021; Li et al., 2023b; Sun et al., 2023). The numerous cardiovascular protective effects of SCU have been found to be directly related to multiple signal pathways and multiple mechanisms of action. By digging deeper into the mechanism of action of drugs, we can discover the real reason behind their magical effects. Increasing research on signal pathways and mechanisms of action has facilitated the development of SCU drugs and guidance for future clinical applications. The US Food and Drug Administration (FDA) has certified SCU as Generally Recognized As Safe (GRAS). Being a BCS Class IV drug, its lower bioavailability affects its efficacy, thus limiting its use to some extent. The bioavailability of SCU after oral administration was very low at 10.67% ± 4.78%, indicating that only a small proportion of SCU can be taken up and used by the body. The reasons for the low bioavailability of SCU after oral administration include low water solubility, unstable chemical properties, intestinal absorption, first-pass effect in the intestine, and first-pass effect in the liver (Wang and Ma, 2018). These factors result in the rapid breakdown and metabolism of SCU in the gut and liver, which reduces its effective concentration in the body and further attenuates the therapeutic effect.

With thousands of years in its development and application, Chinese medicine is widely used in people’s health. The selection of Chinese medicines comes from natural plants, animals, and minerals. This natural selection makes traditional Chinese medicine have multi-target therapeutic characteristics and can comprehensively regulate all aspects of the human body, thereby improving patients’ clinical symptoms. Compared with chemical drugs, Chinese medicines have fewer toxic side effects and are safer and more reliable. Traditional Chinese medicine offers a clear benefit in the treatment of many ailments, and its therapeutic effect has been demonstrated in medical practice. Many of these natural compounds such as naringenin, apigenin, quercetin, ginsenosides, and cinnamaldehyde have shown extraordinary effects on cardiovascular system diseases (Patel et al., 2018; Fan et al., 2020; Heidary Moghaddam et al., 2020; Lu et al., 2022; Thomas et al., 2023).

## 3 Cardiovascular disease-related signal pathways

Under various pathological conditions of the cardiovascular system, as protective events decrease, eventually, regardless of the underlying cause, end-stage cardiac disease will produce the same pathological features of ventricular wall thinning, ventricular dilation, and a sharp increase in interstitial fibrosis. This phenomenon suggests that intracellular signal pathways triggered by different stressors converge on some common targets. The heart is composed of heterogeneous cell groups. The responses of various types of cells to different stimuli are inseparable from the mediation of complex but coordinated signal pathways and the mutual influence of cellular mechanisms, thereby forming multiple physiological responses and pathological processes (Frangogiannis, 2019; Zhang et al., 2022). It can be achieved to develop novel targets and therapeutic approaches for managing cardiovascular illnesses by examining the impact of SCU on various signal pathways.

### 3.1 TGF-β1 signal pathway

Transforming growth factor (TGF) is a cytokine with numerous functions that regulates and takes part in a variety of biological and pathological events in the heart. Saljic et al. (2022), Gu and Liang, (2023), Liang et al. (2022), Ren et al. (2023), Alex et al. (2023), Dong et al. (2023). To protect the heart, the TGF-β signaling system controls apoptosis, autophagy, and antifibrotic activities (Deng et al., 2019; Shen et al., 2020a; Liang et al., 2022). Of these, the most in-depth studies have been conducted on the effects of TGF-β1 on myocardial fibrosis (Garlapati et al., 2023).

**FIGURE 1:**
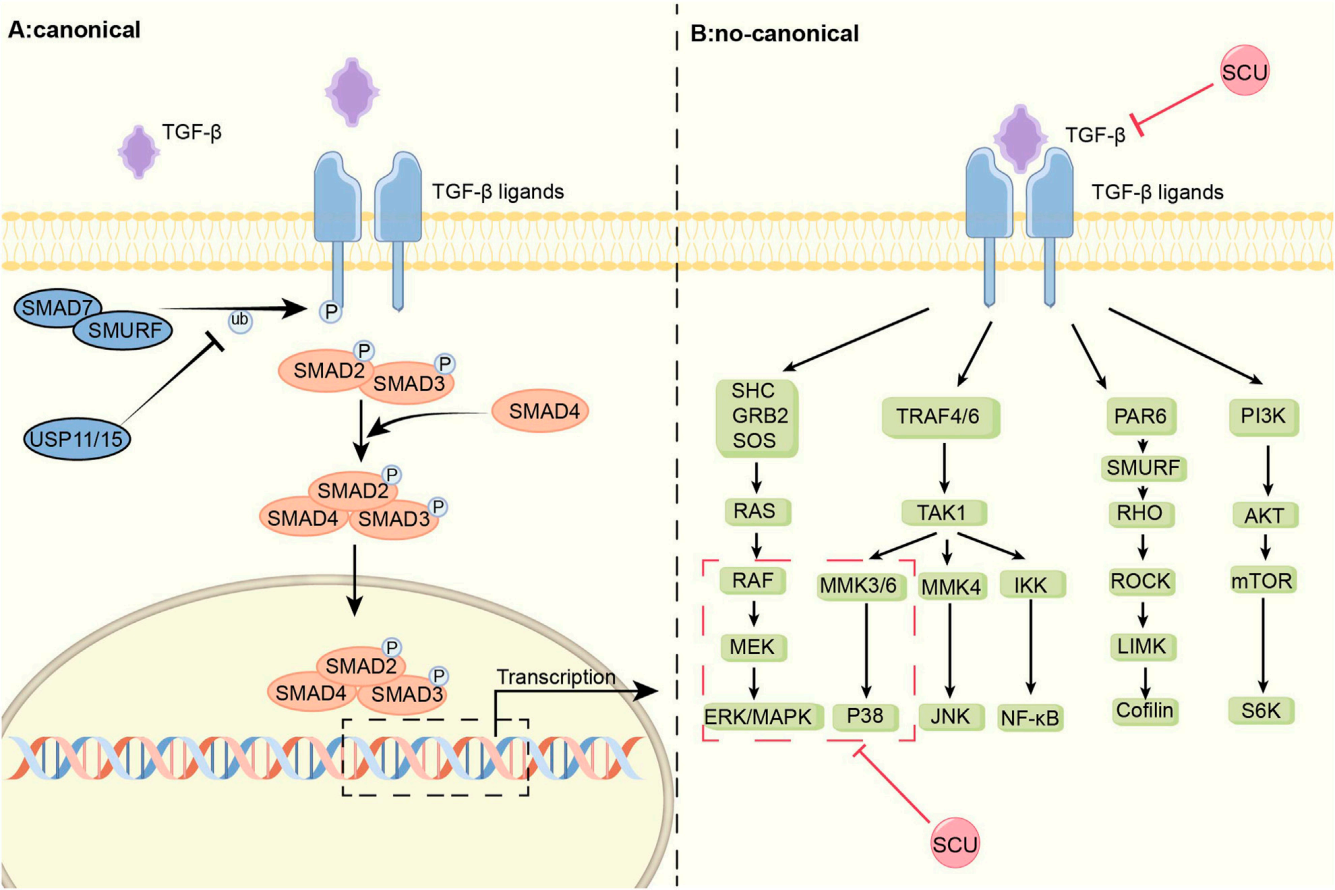
Schematic diagram of the mechanism of SCU regulation of the TGF-β signal pathway. TGF-β signal pathway is divided into classical Smad and non-canonical part. SCU attenuates the expression of TGFβ-1 and inhibits the phosphorylation of p38-MAPK and ERK in the non-classical pathway. ERK: extracellular regulated protein kinases; GRB2:the growth factor receptor-bound protein-2; IKK:IκB kinase; JNK: c-Jun N-terminal kinase; LIMK:LIM-kinases; MEK: mitogen-activated protein kinase kinase; MMK3: medicago MAP kinase 3; Ras: rat sarcoma; Raf: rapidly accelerated fibrosarcoma; RHO: Rho-associated protein kinase; ROCK:Rho kinase; SHC: the adaptor protein SHC; SOS:Son Of Sevenless; S6K: S6 kinase; TRAF:TNF receptorassociated factor.

In rats with myocardial infarction induced by ligation of coronary arteries, Pan et al. (2011) found that SCU prevented the multiplication of cardiac fibroblasts (CFs) and the production of collagen, ultimately reducing interstitial fibrosis by decreasing the expression of FN1 and TGF-β1. It is inferred that SCU may exert its effect on improving the impaired cardiac function in infarcted rats through the TGF-β1 signal pathway. In a different series of Ang II-induced myocardial fibrosis experiments in rats, it was discovered that SCU not only prevented Ang II-induced CFs’ growth and production of collagen as well as downregulated their expression of FN1 and TGF-β1, but also prevented the phosphorylation of both ERK1/2 and p38-MAPK. By controlling the TGF-β1/MAPK signal system, SCU can prevent the formation and progression of cardiac fibrosis. In a study on doxorubicin (DOX)-induced chronic cardiotoxicity, Sun et al. (2023) discovered that SCU inhibited TGF-β1 protein expression and increased pSmad2 levels, reducing the accumulation of collagen and the area of heart fibrosis. Thus, SCU can exert cardioprotective effects through the TGF-β1/Smad2 pathway. In conclusion, SCU can exhibit beneficial effects on the circulatory system by acting on both the traditional and non-classical signal pathways of TGF-β1 (Figure 1).

### 3.2 PI3K/AKT/mTOR signal pathway

The PI3K/AKT/mTOR signal system is a crucial mechanism for controlling cell growth, proliferation, migration, and death and can be crucial for controlling lipid and glucose metabolism (Suber et al., 2018; Senoo et al., 2021; Xiao et al., 2022; Sun et al., 2023). The development of heart-related illnesses is significantly influenced by abnormalities in lipid and glucose metabolism, which are separate risk factors for the cardiovascular system. The PI3K/AKT signal pathway is the primary target of drugs being developed and approved for type II diabetes treatment (Aierken et al., 2022; Fan et al., 2023b). The PI3K/AKT signal system, a key component of the insulin route, controls liver glycogen production, gluconeogenesis, and lipid synthesis to control both glucose balance and lipid synthesis (Huang et al., 2018b; Petersen and Shulman, 2018).

In the cytotoxicity experiment, Zhou et al. discovered that SCU increased the expression of p-AKT, p-mTOR, and p62 while down-regulating the expression of Beclin 1 and LC3-II. This resulted in a reduction in the rate of cell death and a restoration of cell viability (Zhou et al., 2022b). In this work, it was shown that SCU might protect cells by inhibiting the autophagy process via the PI3K/AKT signal pathway. In a different study, Fan et al. (2017) discovered that SCU increased the expression of the proteins Nrf2, HO-1, PI3K, AKT, and NQO1 in rat livers with non-alcoholic fatty liver disease to reduce oxidative damage and enhance lipid metabolism. It was deduced that PI3K/AKT phosphorylation and consequent Nrf2 transfer were necessary for SCU’s anti-hyperlipidemic action. Xu et al. (2021) found in diabetic cardiomyopathy (DCM) mice, SCU improved cardiac function by preventing the decline of p-AKT and increasing the subsequent Nrf2 translocation with HO-1 protein expression in diabetic mouse cardiomyocytes. It can be inferred that the PI3K/AKT/mTOR signal pathway played a role in how protective SCU was for cardiomyocytes. Fu et al. (2019) found in an apoptosis-inducing assay in human aortic endothelial cells that SCU increased the cell viability of post-injury human aortic endothelial cells by elevating the levels of PI3K, P-AKT, and P-FOXO3A and that PI3K inhibitors could attenuate this promotion.

**FIGURE 2:**
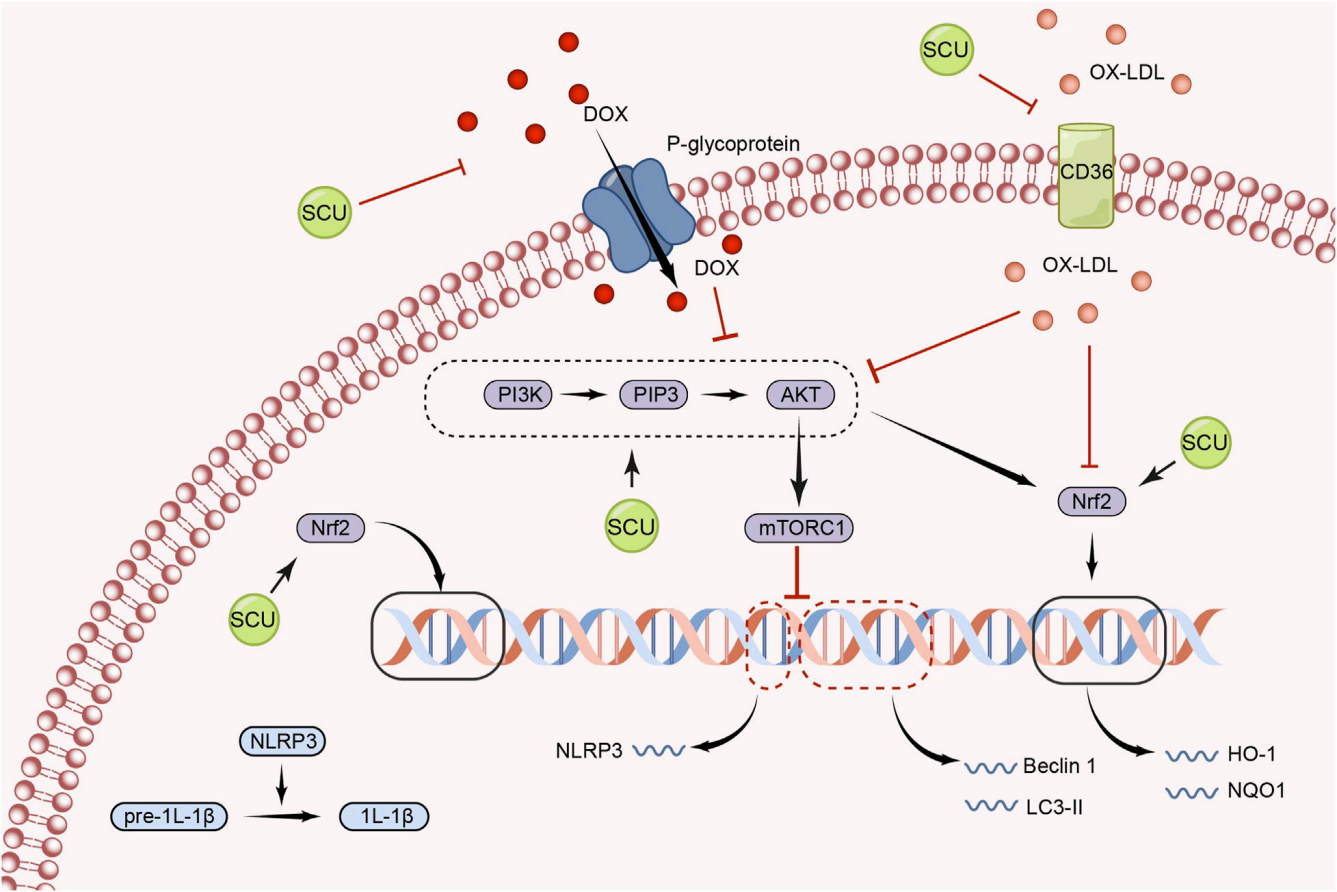
Schematic representation of the mechanism by which SCU regulates the PI3K/AKT/mTOR signal pathway to attenuate inflammation, apoptosis, and oxidative responses. CD36: the scavenger receptor B2; IL-1β: Interleukin-1beta; LC3: light chain 3; NQO1: NAD(P)H:quinone oxidoreductase 1; ox-LDL: oxidized low-density lipoprotein; PIP3: phosphatidylinositol 3,4,5-trisphosphate.

Numerous inflammatory processes are mediated by the NLRP3 inflammasome (NLRP3), which is activated by mTOR signal (Dai et al., 2019; Yang et al., 2019; Marín-Aguilar et al., 2020; Ye et al., 2020; Chen et al., 2021). Xu et al. (2020) found that SCU exerted a role in inhibiting NLRP3 activation and thus attenuating the inflammatory response by increasing AKT phosphorylation and inhibiting mTORC1 activity in experiments in which acute myocardial I/R injury induced H9c2 damage. Furthermore, this study found that SCU-mediated inhibition of mTORC1 and activation of NLRP3 could be abolished by gene silencing of AKT by siRNA. In conclusion, SCU has the ability to protect the cardiovascular system by activating the PI3K/AKT/mTOR signal pathway (Figure 2).

### 3.3 Nrf2/Keap/ARE signal pathway

Regarding redox homeostasis, DNA repair, iron homeostasis, cell proliferation, and other processes, nuclear factor erythroid 2-related factor 2 (Nrf2) is among the most active activators of transcription in the Cap ‘n ‘Collar family. One of the most vital cellular routes is the Nrf2/Keap/ARE signal pathway. This mechanism reduces oxidative stress and eliminates excess ROS to maintain redox equilibrium *in vivo* (Chen, 2022).

**FIGURE 3:**
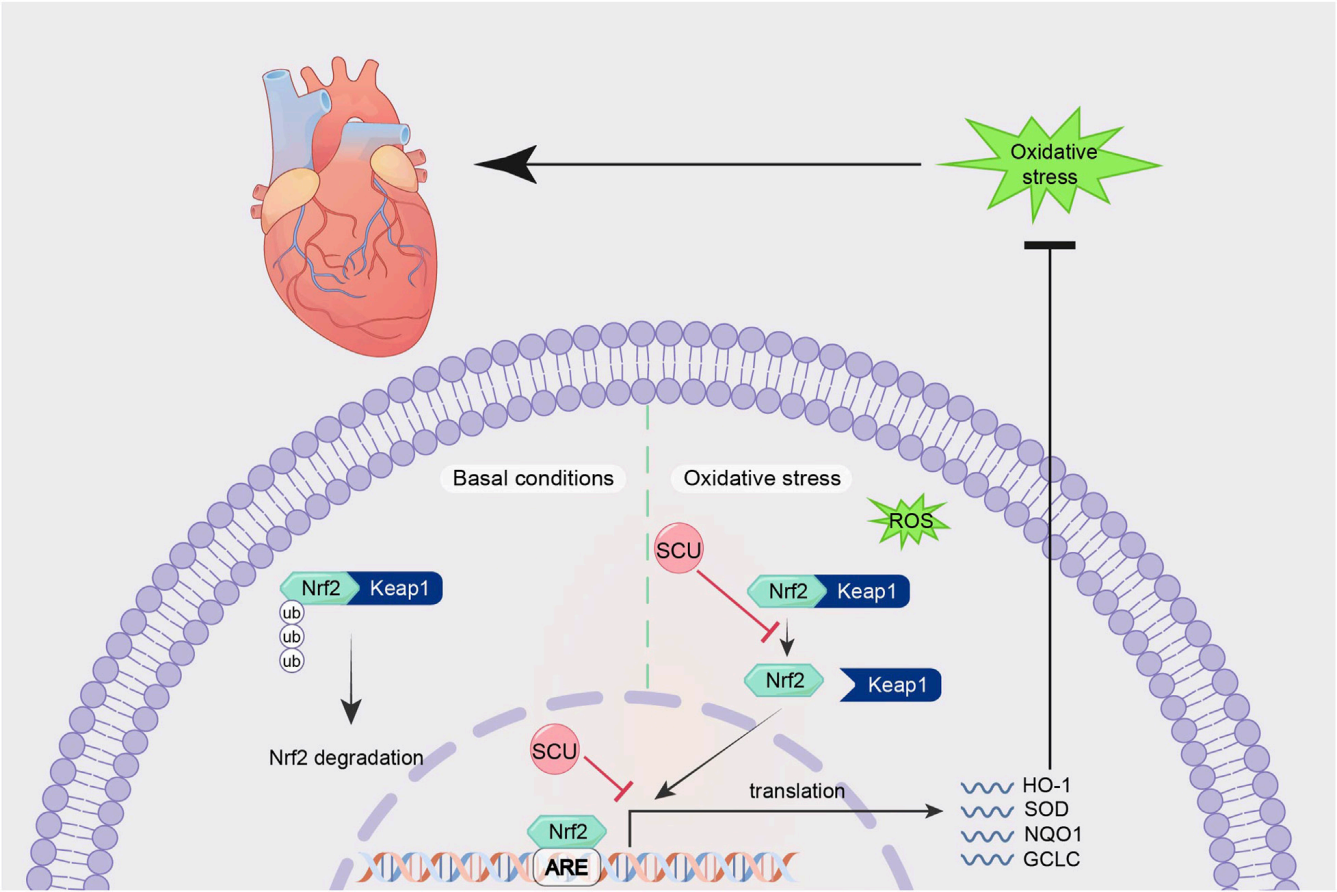
Schematic mechanism of SCU regulation of Nrf2/Keap/ARE signal pathway. SCU upregulates Nrf2, NQO-1, and HO-1 mRNA expression and downregulates Keap1 mRNA expression to alleviate oxidative stress. Keap1:Recombinant Kelch Like ECH Associated Protein 1; ROS: reactive oxygen species.

By promoting the expression of Nrf2, NQO-1, and HO-1 and suppressing the expression of Keap1 mRNA in the hearts of diabetic mice, Huo et al.'s research in a mouse model of type 2 diabetes revealed that SCU plays an essential part in reducing oxidative damage and the severity of type 2 diabetes-induced cardiac complications (Huo et al., 2021). SCU significantly elevated the expression of the proteins Nrf2 and HO-1 and reduced oxidative damage in mice with STZ-induced DCM, according to Xu et al.'s findings (Xu et al., 2021). It suggests that SCU may exert cardioprotective effects against diabetic injury through the Nrf2/Keap/ARE signal pathway. Fan et al. (2017) in an experiment to induce hyperlipidaemia in rats, found that SCU attenuated oxidative damage by increasing the expression of Nrf2, HO-1, PI3K, and AKT proteins, thereby improving serum and liver lipid metabolism levels. This suggests that through the Nrf2/Keap/ARE signal pathway, SCU can contribute to improved lipid metabolism and anti-hyperlipidemia (Figure 3).

### 3.4 NOTCH signal pathway

NOTCH signal is an event that regulates differentiation, proliferation, and apoptosis through cell-to-cell interactions. In the growth, maturation, and restoration of the heart, NOTCH signal is crucial (Zhou et al., 2022a).

**FIGURE 4:**
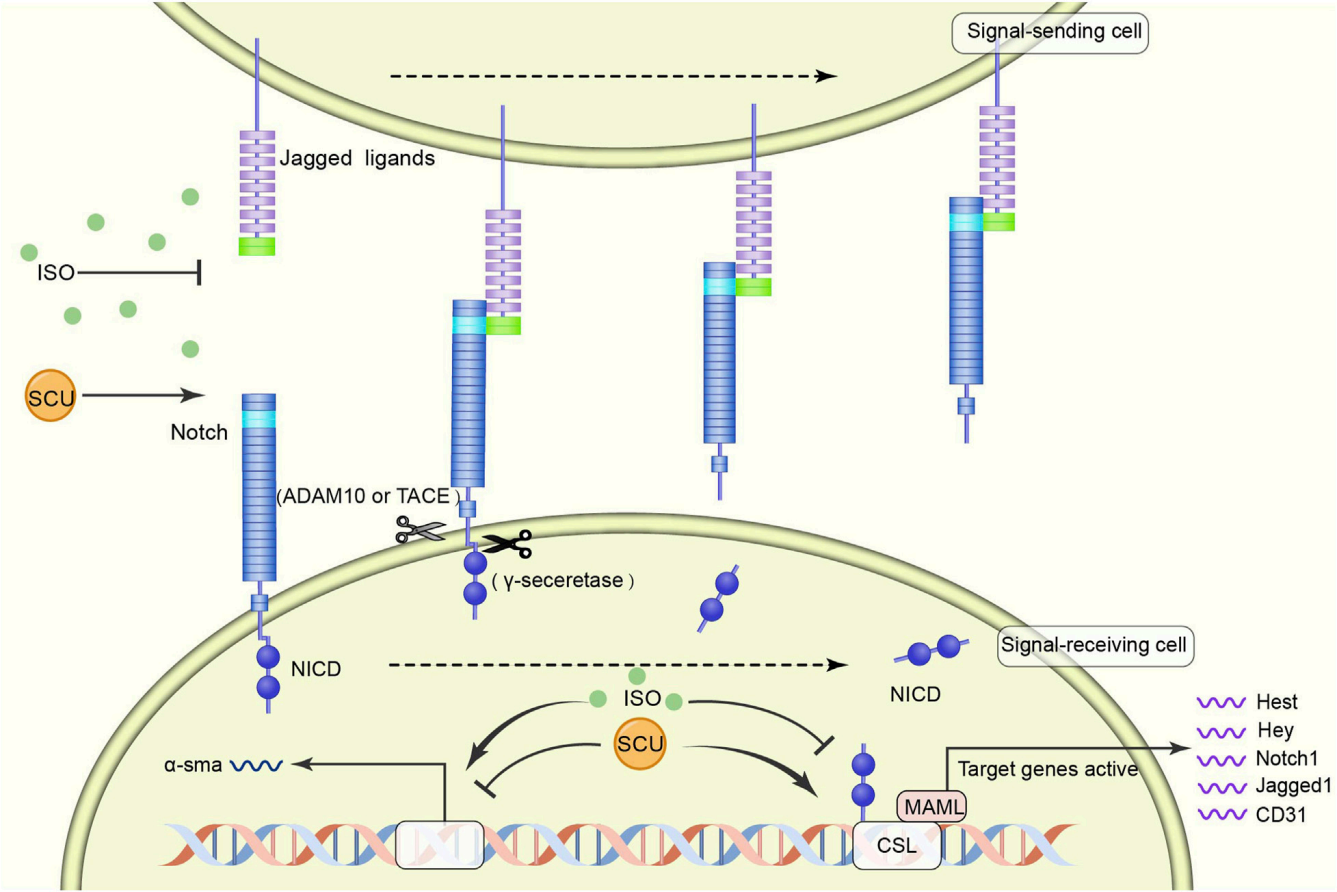
Schematic diagram of the regulatory mechanism of SCU on NOTCH signal pathway. SCU blocks the inhibitory effects of harmful factors on the pathway and increases the expression of CD31, Notch1, Jagged1, and Hes1. ADAM10: transmembrane endopeptidase ADAM10; CD31: platelet/endothelial cell adhesion molecule-1; CSL (CBF1, Suppressor of Hairless, Lag-1) transcription factor; MAML: mastermind-like transcriptional coactivators; NICD: Notch intracellular domain; *a*-sma:α smooth muscle actin.

Zhou et al. (2014) found in an experimental model of myocardial fibrosis in rats that SCU inhibited the development of myocardial fibrosis by reversing the induction of increased *a* smooth muscle actin expression and decreased CD31, Notch1, Jagged1, and Hes1 expression. It suggests that SCU can exert cardioprotective effects against myocardial fibrosis through the NOTCH pathway (Figure 4).

### 3.5 eNOS/cGMP/PKG signal pathway

In recent years, the eNOS/cGMP/PKG signal pathway has been considered an important target for therapies such as regulating blood pressure, attenuating IR injury, and delaying heart failure (Anwar et al., 2017; Kolijn et al., 2021; Park et al., 2022). Additionally, the eNOS/cGMP/PKG signal route is crucial for controlling blood pressure and vascular endothelial function (Zhang et al., 2023).

**FIGURE 5:**
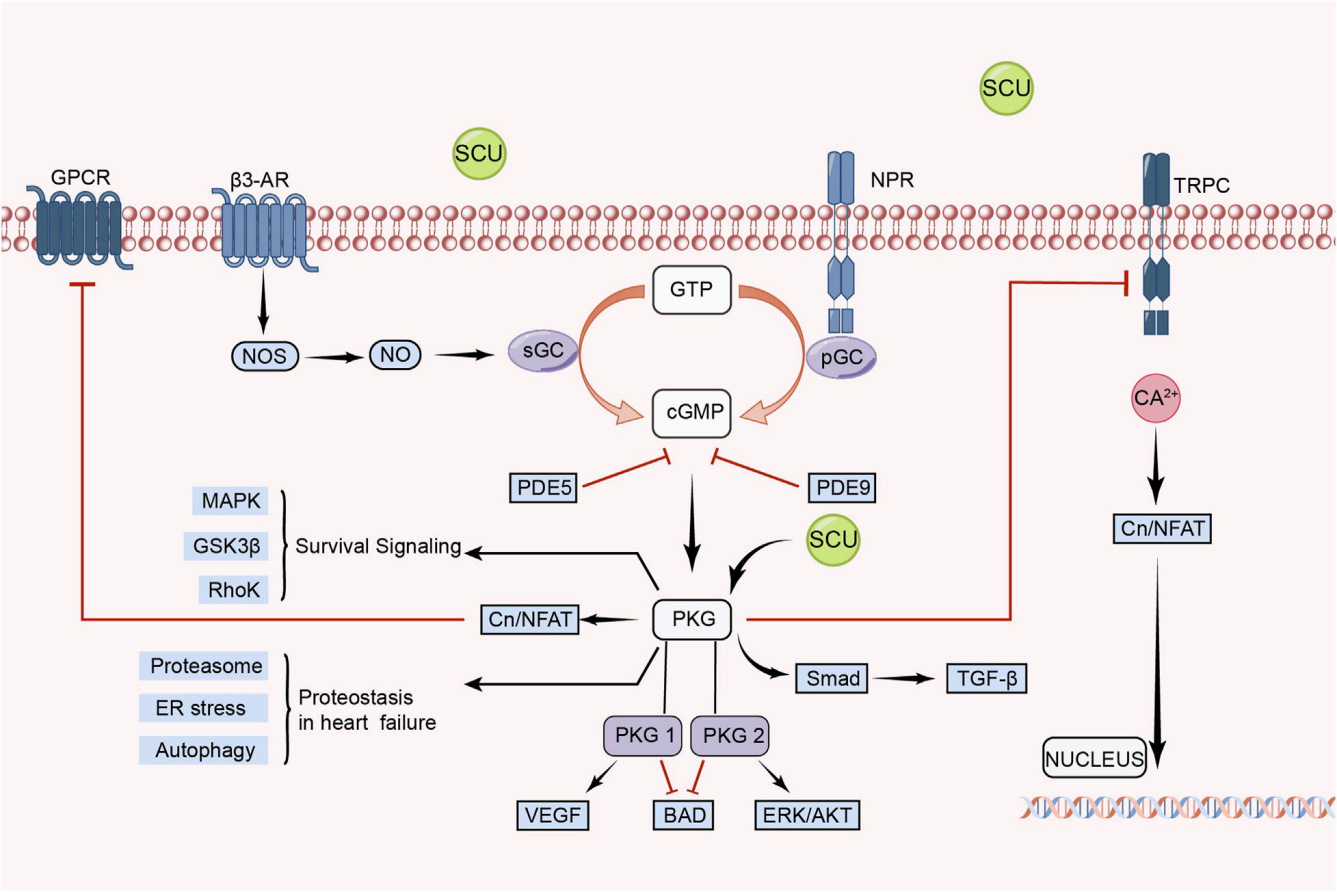
Schematic representation of SCU regulation of eNOS/cGMP/PKG signal pathway. SCU exerts its protective effect on damaged cells mainly by activating PKG. CN: Calcineurin; GPCRs: G protein-coupled receptors; GSK3-β: glycogen synthase kinase-3β; GTP: guanosine triphosphate; NFAT: nuclear factor of activated T cells; NO: Nitric oxide; NOS: NO synthase; NPR: Neuropeptide receptor; PDE: Phosphodiesterase; PGC: Peroxisome proliferator-activated receptor-γ coactivator; sGC: soluble guanylate cyclase; TRPC: Transient Receptor Potential Canonical; VEGF: vascular endothelial-derived growth factor; β3-AR: β3-adrenergic receptor.

Li et al. (2015) found in an experimental model of myocardial ischemia-reperfusion (MIR)in rats that SCU was able to exert an anti-MIR injury effect by increasing the levels of p-VASP Ser239 in rat cardiac tissue and serum. p-VASP Ser239 is a marker of PKG activation. Therefore, the protective effect of SCU against MIR injury is related to the PKG pathway. They also performed human cardiac microvascular endothelial cells injury experiments. It was discovered that SCU might have a positive impact on hypoxia reoxygenation (HR)-injured endothelial cells by reversing the decrease in PKG-I, PKG-I phosphorylation, and PKG-I mRNA after HR injury and, concurrently, raising p-VASP Ser239 and the ratio of p-VASP Ser239 to total VASP. Chen et al. (2015) found that SCU exerted endothelium-dependent relaxation and attenuated endothelial damage by increasing pVASP protein levels in HR-induced endothelial dysfunction in isolated rat CA. This experiment demonstrated that SCU can perform vascular endothelial protection through the PKG pathway. In conclusion, SCU can protect the cardiovascular system by activating the eNOS/cGMP/PKG signal pathway (Figure 5).

### 3.6 PINK1/Parkin signal pathway

The PINK1/Parkin signal pathway is closely related to “mitophagy" (Wang et al., 2021a). An important part of the metabolism of heart energy is played by mitochondria. However, too much ROS generation brought on by mitochondrial malfunction destroys cardiomyocytes and causes a number of cardiovascular disorders. The injured mitochondria in this situation need to be removed. Mitophagy is crucial for preserving heart homeostasis. Cardiac homeostasis is inseparable from mitochondrial autophagy, which is inseparable from the PINK1/Parkin signal pathway.

**FIGURE 6:**
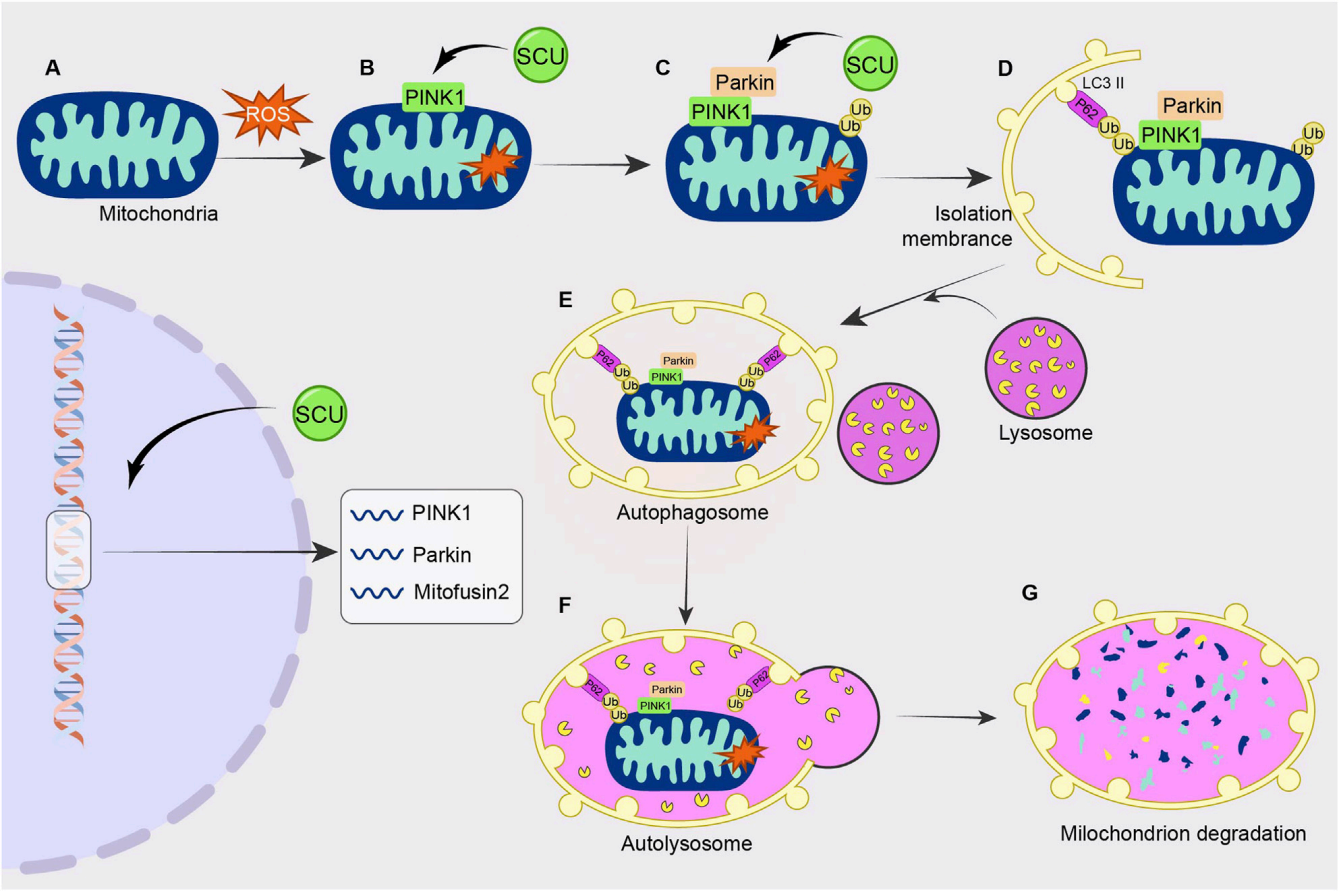
Schematic diagram of the regulatory mechanism of SCU on PINK1/Parkin signal pathway. SCU increases PINK1 levels and promotes the expression of PINK1, Parkin, and Mitofusin2 to attenuate high glucose-induced cellular injury.

Xi et al. (2021) found in human umbilical vein endothelial cells (HUVECs) injury experiments that SCU reduced the expression of P62 and apoptotic proteins Cyt. C, cleaved caspase3 by elevating the High glucose-induced reduced levels of PINK1. Meanwhile, SCU promoted the expression of PINK1, Parkin, and Mitofusin2. Thus, SCU exerts a cell viability-enhancing and vascular endothelial protective effect on HUVECs by activating autophagy and attenuating apoptotic pathways. This study confirmed that SCU exerts a protective effect on vascular endothelium through the PINK1/Parkin signal pathway (Figure 6).

### 3.7 JAK2/STAT3 signal pathway

The JAK/STAT signal pathways involve biological functions such as cell apoptosis, cell cycle, and stem cell homeostasis. The JAK2/STAT3 pathway is one of the JAK/STAT pathways (Verhoeven et al., 2020; Xin et al., 2020). Previous studies have demonstrated that the JAK2/STAT3 pathway can potentially alleviate oxidative stress, apoptosis, and other mechanisms that contribute to mitigating myocardial IR injury (Mahdiani et al., 2022).

**FIGURE 7:**
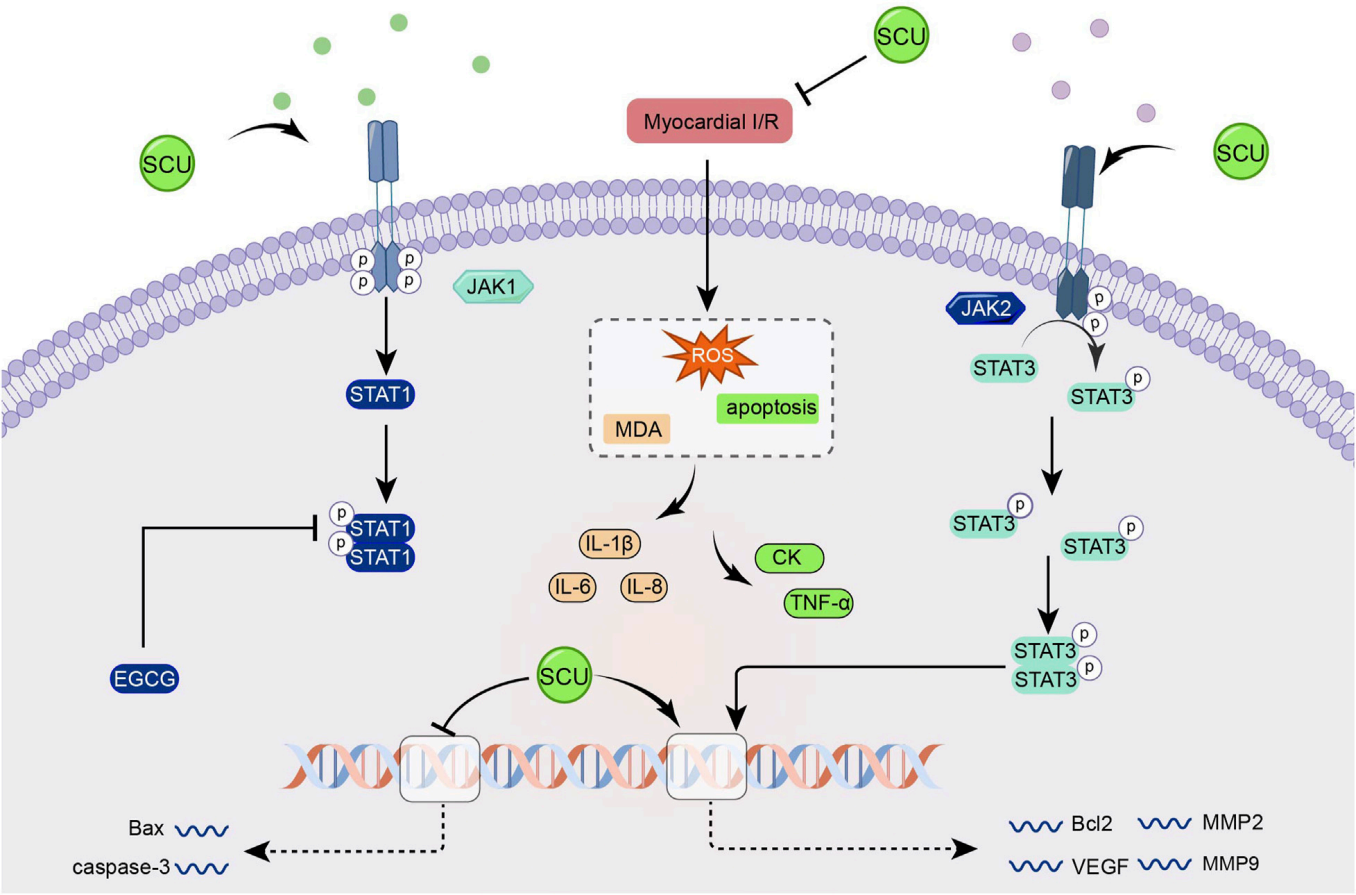
Schematic representation of SCU attenuating I/R damage-induced oxidative stress and apoptosis by enhancing the JAK2/STAT3 pro-survival signal pathway. CK: Creatine kinase; EGCG: epigallocatechin-3-gallate; MDA: Malondialdehyde; MMP2: matrix metallopeptidase 2; MMP9: matrix metallopeptidase 9.

Wang et al. (2016) found that SCU increased the expression of Bcl2, VEGF, MMP2, MMP9, and SOD, attenuated the expression of Bax and caspase-3 and the level of MDA through the JAK/STAT3 signal pathway and exerted cardioprotective effects in the experiments on I/R-injured H9c2 (Figure 7).

### 3.8 CaMKII signal pathway

In the cardiovascular system, calcium signal is central to cardiac physiology and is closely related to the contraction and diastole of cardiac tissue and endovascular myocytes (Nattel et al., 2020; Chen et al., 2022b). Dysregulated calcium signals can lead to abnormal blood pressure, cardiac hypertrophy, heart failure, and other diseases. (Beckendorf et al., 2018; Basu et al., 2019; Luczak et al., 2020).

**FIGURE 8:**
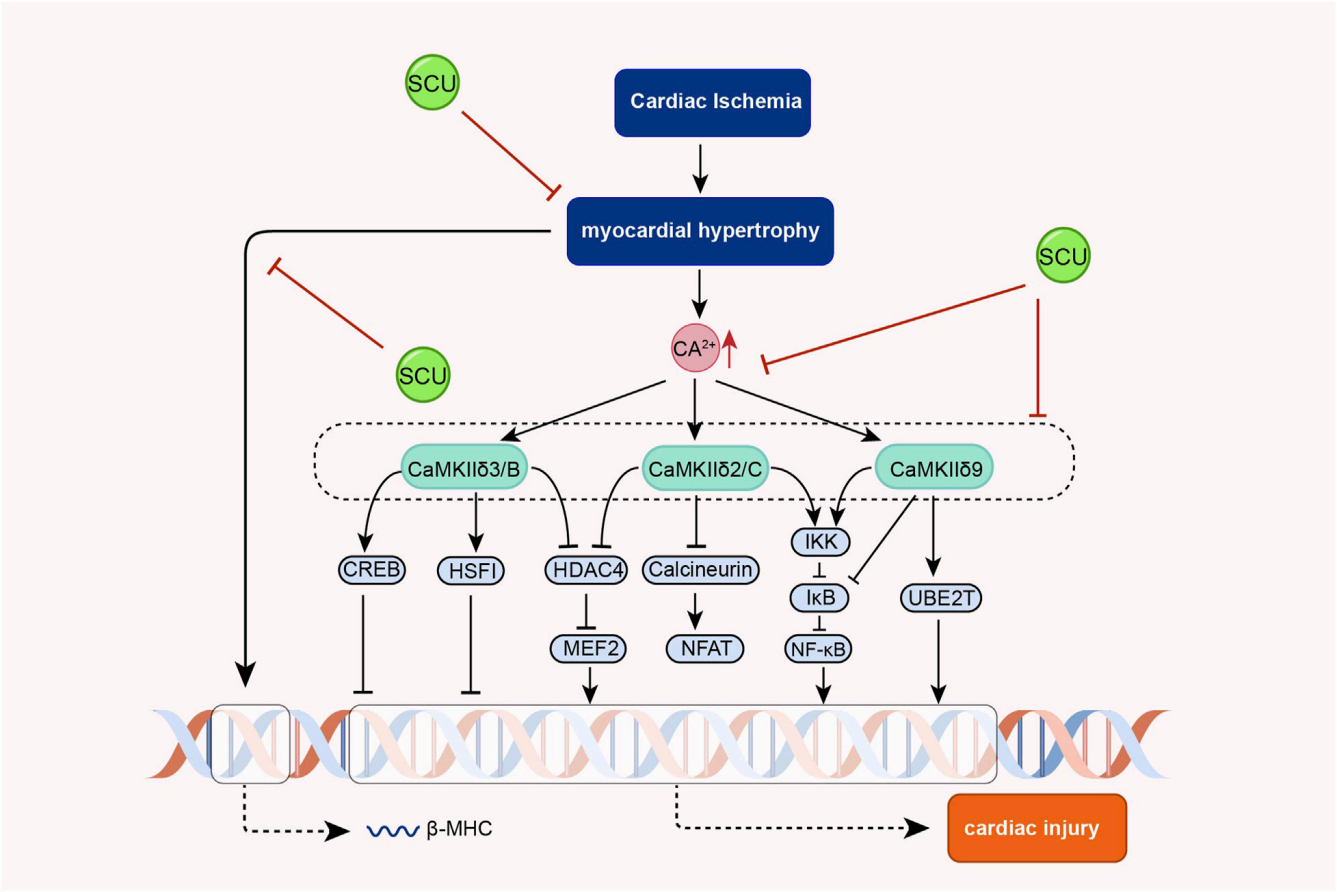
Schematic representation of the mechanism of SCU inhibition of Ca^2+^-mediated CaMKII signal pathway against cardiac hypertrophy. CREB: cAMP responsive element binding protein; HDAC4 (histone deacetylase 4; HSF: hepatocyte-stimulating factors; IkB: Inhibitor-kB; MEF2: myocyte Enhancer Factor 2.

Earlier, Pan and others found that SCU exerted endothelium-independent vasorelaxation by inhibiting extracellular calcium inward flow in isolated rat aortas in experiments in which noradrenaline bitartrate induced aortic constriction in rats and that this effect was independent of vdcs (Pan et al., 2008). Subsequently, Pan et al. (2010) found that SCU exerted its anti-cardiac hypertrophic influence by inhibiting the increase of intracellular calcium and calcineurin and inhibiting the expression of calcineurin in experiments with phenylephrine-induced hypertrophy of neonatal rat cardiomyocytes, and a model of pressure overload-induced cardiac hypertrophy in mice. In further AB mouse experiments, SCU inhibited phosphorylated CaMKII that was elevated after AB treatment. However, phosphorylated CaMKII is the active form of CaMKII. Thus, the team demonstrated, by means of a progressive research approach, that SCU can exert significant anti-cardiac hypertrophic effects by inhibiting the Ca^2+^-ediated CaMKII signal pathway (Figure 8).

### 3.9 TLR4/MyD88/NF-κB signal pathway

The classical TLR4/MyD88/NF-κB signal route is involved in activating processes such as inflammatory responses, oxidative stress, and immune regulation in the organism (Shen et al., 2020b; Guo et al., 2021; Liu et al., 2022). In the cardiac system, the TLR4/Myd88/NF-κB pathway has a regulatory role in hypertension and a protective effect on the heart (Kim et al., 2020; Yang et al., 2020). By reducing oxidative stress, inflammation, and apoptosis, the TLR4/NF-κB signal pathway may reduce hyperglycemia and diabetes-induced cardiomyopathy (Yao et al., 2021).

**FIGURE 9:**
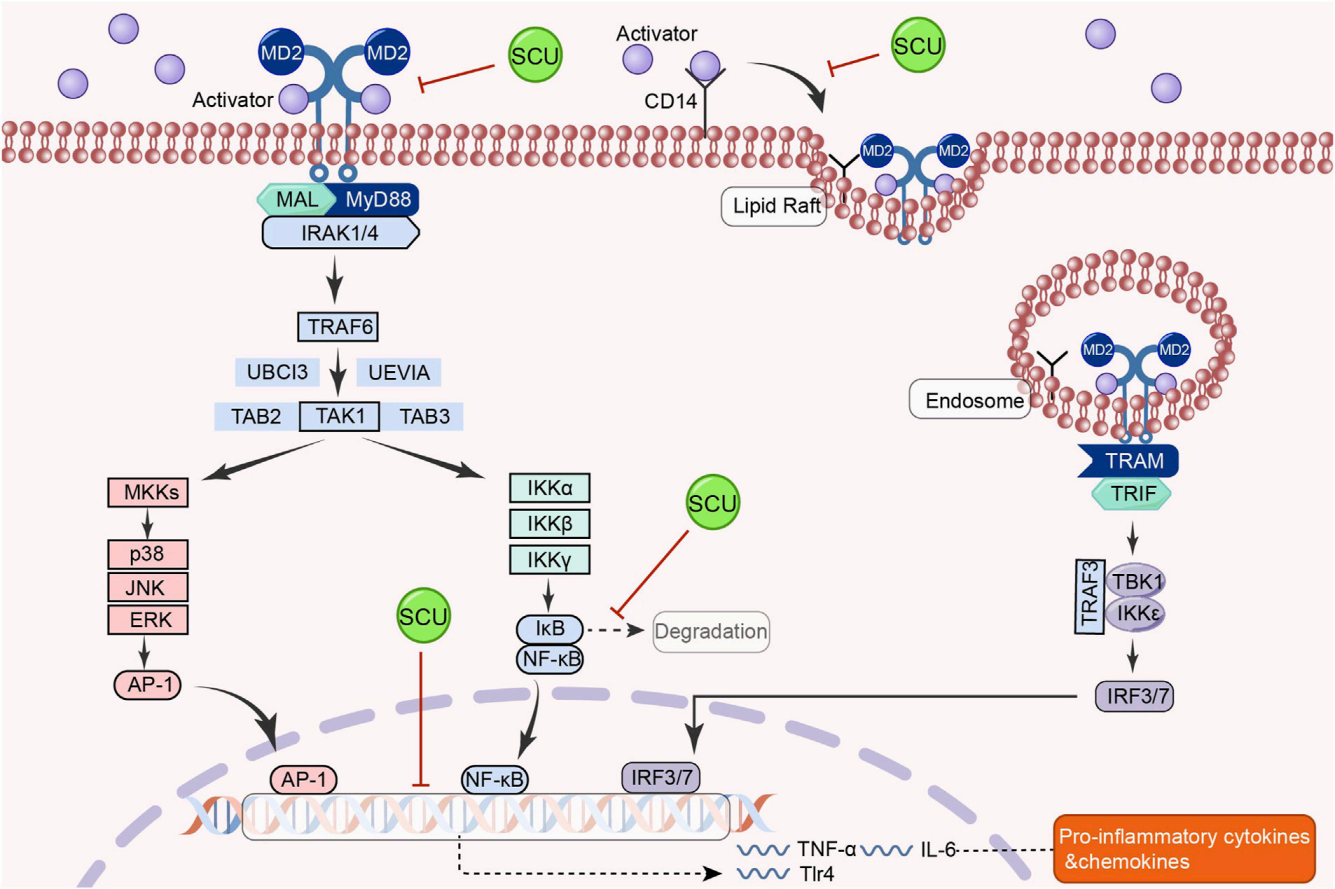
Schematic representation of the mechanism by which SCU inhibits the TLR4/Myd88/NF-κB signal pathway to attenuate the inflammatory response in the heart. AP-1: activator protein-1; CD14: co-receptor for toll-like receptors; IRAK1: interleukin-1 receptor-associated kinase 1; IκB: inhibitor-kB; IRF3: Interferon regulatory factor 3; IRF7: interferon regulatory factor 7; MAL:myD88-adapter-like; MD2: myeloid differentiation protein 2; MKK: mitogen-activated protein kinase kinase; TAB2: TGF-β-activated kinase 1 binding protein 2; TAB3: TGF-β-activated kinase 1 binding protein 3; TAK1: TGF-β-activated kinase 1; TBK1: the TANK-binding kinase 1; TRAM: TLR4 recruits TRIF-related adaptor molecule; TRIF: Toll/IL-1R domain-containing adaptor-inducing IFN-β; UBCI3: E2 ubiquitin conjugating enzyme; UEVIA:E2 ubiquitin conjugating enzyme.

In a rat model of hypertension, Chen et al. (2013) discovered that SCU could have tissue-protective and antihypertensive effects by upregulating Mcl1 and downregulating inflammatory and apoptotic factors like TLR4, NF-κB, p65, TNF-α, IL-1β, IL-18, Bax, and cleaved-caspase-3 p17. In addition, Huo and others found that SCU inhibited the increase of cardiac inflammatory markers in diabetic mice, such as TLR4, MyD88, NF-κB, and IL-6, through the TLR4/MyD88/NF-κB signal pathway, as well as inhibited the increase in the protein distribution of NF-κB and TNF-α and the decrease in the protein distribution of IKKβ in the diabetic cardiac immunohistochemical sections in their experiments on the type 2 diabetes mellitus model (Huo et al., 2021). SCU reduces the heart damage caused by type 2 diabetes by activating this signal route. The above studies demonstrated that SCU acts on the TLR4/MyD88/NF-κB signal pathway to exert antihypertensive and antidiabetic effects (Figure 9).

### 3.10 cGAS-STING signal pathway

The cGAS-STING signal pathway was originally recognized for its role in immune defense due to its immune recognition of cytoplasmic DNA (Zhang et al., 2020b). As an emerging hot pathway in recent years, it can have a considerable impact on the cardiovascular system (Wang et al., 2020; Oduro et al., 2022; Luo et al., 2023). Some of these studies have found that the cGAS-STING signal pathway may be a critical therapeutic target for improving the prognosis of myocardial infarction and ischaemic reperfusion injury (Rech et al., 2022; Lv et al., 2023).

Li et al. (2023b). found that intraperitoneal injection of SCU attenuated I/R-induced apoptosis of cardiomyocytes in mice while improving I/R-induced diminished cardiac function in an *in vivo* experiment in mice with cardiac I/R injury. Moreover, SCU reduced the expression of cGAS, STING, and cleaved caspase3 in I/R injury-induced cardiac tissues while increasing the Bcl2/Bax ratio. This experiment suggests that the effect of SCU in improving cardiac function in mice may be related to the cGAS-STING signal pathway. Then, in an *in vitro* experiment of H/R-induced H9c2 cell injury, Li et al. found that H/R led to apoptosis of H9c2 cells while increasing the expression levels of cGAS, STING, and cleaved caspase3 and decreasing the Bcl2/Bax ratio. This phenomenon can be reversed by SCU and cGAS inhibitors. Thus, this study suggests that SCU inhibits myocardial apoptosis induced by activation of the cGAS-STING signal pathway, thereby exerting a cardioprotective effect.

## 4 Mechanism of action of SCU on CVD

### 4.1 Suppression of the inflammatory reaction

Atherosclerosis, diabetic cardiomyopathy, myocardial infarction, and myocardial ischemia-reperfusion injury are only a few examples of cardiovascular illnesses influenced by inflammatory reactions (Chistiakov et al., 2017; Fredman and MacNamara, 2021; Goswami et al., 2021; Avagimyan et al., 2022). As the population ages and living standards improve, physiopathological factors such as aging, hyperglycemia, and hyperlipidemia exacerbate the development of an inflammatory response in the cardiovascular system, ultimately leading to heart failure (Chistiakov et al., 2017; Goldfine and Shoelson, 2017; Adamo et al., 2020). To prevent and treat CVD, it is crucial to effectively reduce the inflammatory response. According to a few studies, SCU has cardioprotective properties by reducing inflammatory reactions.

Huo et al. (2021) found that SCU could attenuate cardiac histopathological changes by decreasing high fat diet/streptozotocin (HFD/STZ)-induced upregulation of TLR4, Myd88, NF-κB, IL- 6, and TNF-α and by increasing HFD/STZ-induced downregulation of IkBβ mRNA expression in a mouse model of type 2 diabetes mellitus. It suggests that SCU may exert cardioprotective effects by reducing cellular damage by inhibiting inflammatory responses. In another study, SCU could exert an inhibitory effect on the activation of NLRP3 through activation of AKT and inhibition of mTORC1, which in turn exerted a cardioprotective effect (Xu et al., 2020). In addition, Huang et al. found in isoproterenol (ISO)-induced myocardial infarction in rats that SCU could play a role in attenuating cardiac injury by decreasing the expression of myocardial inflammatory cytokines, such as gelatinase-associated lipid transport protein, NF-κB, IL-1β, and IL-6, in neutrophils induced by ISO(Huang et al., 2018a). In other cases, Xu et al. (2021) found in streptozotocin (STZ)-induced DCM in small mice that SCU attenuated myocardial damage in diabetic mice by inhibiting the activation of NLRP3, the release of proinflammatory cytokines, and the nuclear translocation of NF-κB. In summary, SCU can exert cardioprotective effects by suppressing the inflammatory response.

### 4.2 Mitigation of oxidative stress

Cardiovascular illnesses like hypertension, atherosclerosis, and other ischemic heart diseases are influenced by oxidative stress (Guzik and Touyz, 2017; Kibel et al., 2020). Moreover, excessive oxidative stress accelerates the rate of cardiovascular system aging as the body ages (Kibel et al., 2020). Oxidative stress is also inextricably linked to hyperlipidemia, diabetes, and metabolism-related cardiac complications (Zhang et al., 2020a; Fuller et al., 2020; Tao et al., 2021). Therefore, modulation of oxidative stress is essential to mitigate CVD. Some studies have found that SCU can exert cardiovascular protection through antioxidant responses (Table 1).

**TABLE 1:** Summary of experiments on the alleviation of oxidative stress by SCU.

Experimental model	Mechanism	Effect	Ref
db/db mice	Promots the Nrf2/HO-1 signal pathway	Reduces oxidative stress response, exerts hypoglycemic effect	Liu et al. (2019)
non-alcoholic fatty liver disease rats	Promotes PI3K/AKT signal pathway, promotes Nrf2 nuclear translocation, increases HO-1, NQO1 expression	Reduces oxidative stress, lowers blood lipids	Fan et al. (2017)
HFD/STZ-induced type 2 diabetic mice	Promots the Nrf2/Keap1 signal pathway	Reduces oxidative stress and resists type 2 diabetes-induced cardiac damage	Huo et al. (2021)
H_2_O_2_-injured HUVECs	Reduces ROS and promotes SOD1 and Nox4 mRNA expression	Reduces oxidative stress, protects vascular endothelium	Mo et al. (2018)
HFD diet male rats	Increases SOD and NO and decreases MDA	Alleviates oxidative stress, reduces serum TC, TG and LDL-C, and resists atherosclerosis	Mo et al. (2018)
DOX-induced cytotoxicity of H9c2, CFs and HUVECs	Reduces ROS and MDA, increases SOD activity	Reduces oxidative stress, protects heart tissue	Zhou et al. (2022b)
DOX-induced cardiotoxicity in male rats	Reduces LDH activity and MDA	Reduces cTnT concentration, increases LVEF and LVFS, and reverses cardiac tissue damage	Sun et al. (2017)
ISO induced myocardial infarction in rats	Increases SOD activity, CAT activity, GSH, decreases MDA, iNOS	Reduces oxidative stress and reduces myocardial infarction	Huang et al. (2018a)
I/R-induced damage to H9C2	JAK2/STAT3 signal pathway, reduces SOD and increases MDA	Reduces oxidative stress and protects against myocardial I/R injury	Wang et al. (2016)
STZ-induced DCM in mice	Increases SOD activity, CAT activity, GSH Px activity, reduces MDA and ROS, and activates Nrf2/HO-1 pathway	Alleviates oxidative stress, reduces cardiac damage and fibrosis	Xu et al. (2021)

CAT,catalase; cTnT, cardiac troponin-T; GSH, glutathione; LDH, lactate dehydrogenase; LVEF, left ventricular ejection fraction; LVFS, left ventricular fractional shortening.

### 4.3 Regulation of apoptosis

Apoptosis, also known as programmed cell death, can mediate many cardiac pathologies such as heart failure, myocardial infarction, ischaemia-reperfusion injury, diabetic cardiomyopathy, and vascular endothelial injury (Cheng et al., 2020; Li et al., 2021; Liao et al., 2022; Liu et al., 2023). Promoting apoptosis exacerbates CVD, whereas limiting apoptosis exerts a cardioprotective effect. Recent research has revealed that SCU affects the apoptotic process, which could lead to the development of novel therapies for the treatment of connected diseases (Table 2).

**TABLE 2:** Summary of experiments on SCU regulation of apoptosis.

Experimental model	Mechanism	Effect	Ref
HFD rats, AngII-induced human aortic endothelial cells apoptosis	Hippo-FOXO3A and the PI3K/AKT signal pathway	Inhibits endothelial cell apoptosis and resists atherosclerosis	Fu et al. (2019)
HFD/STZ-induced type 2 diabetes in mice	Downregulates the expression of Bax, Cyt-c, Caspase-9, Caspase-3 and Parp 1 genes, and upregulates the expression of Bcl-2 gene	Inhibits cardiomyocyte apoptosis	Huo et al. (2021)
High glucose-induced injury in HUVECs	Increases Bcl-2, reduces Bax, promotes Cyt-C and Caspase-3 expression	Inhibits endothelial cell apoptosis	Xi et al. (2021)
Acute myocardial ischemia-reperfusion -induced injury of H9c2 cells	Increases Beclin-1 protein and upregulates LC3B II/I ratio	Inhibits cardiomyocyte apoptosis and promotes autophagy	Xu et al. (2020)
DOX-induced damage to H9c2 cells, CFs and HUVECs	Reduces Bax, p53, downregulates Bax/Bcl-2 ratio, inhibits expression of caspase 3 pro-apoptotic proteins, and promotes expression of Bcl-2 anti-apoptotic proteins	Inhibits apoptosis	Zhou et al. (2022b)
High-fat, high-sugar diet-induced type 2 diabetic cardiomyopathy	Inhibits the activity and expression of caspase-3, caspase-8, caspase-9 and caspase-12, inhibits the mRNA and protein expression of Bax and Cyt-C, and promotes the mRNA and expression of Bcl- 2	Inhibits cardiomyocyte apoptosis	Su et al. (2022)
ISO-induced myocardial infarction in rats	Inhibits the expression of Bax, P53, Caspase-3, Caspase-9 and Cyt-C	Inhibits cardiomyocyte apoptosis	Huang et al. (2018a)
I/R-induced H9C2 injury	Promotes JAK2/STAT3 pro-survival signal, increases STAT3, and inhibits Bcl2, VEGF, MMP2 and MMP9 expression	Inhibits cardiomyocyte apoptosis	Wang et al. (2016)
DOX-induced chronic cardiotoxicity in rats	Inhibites Bax, p53, cleavedcaspase3 expression, downregulates Bax/Bcl2 and cleaved caspase3/caspase3 ratio	Inhibits cardiomyocyte apoptosis	Sun et al. (2023)

Cyt-c, cytochrome c; FOXO3A, Forkhead box class O3A; HG, high glucose; LC3B, light chain 3B.

### 4.4 Vascular endothelial protection

Endothelial cells make up the vascular endothelium. The regulation of vasodilatory tone and angiogenesis are two functions that endothelial cells do (Alvandi and Bischoff, 2021; Trimm and Red-Horse, 2023). As a result, endothelial function plays a key role in the development of numerous illnesses, including hypertension, atherosclerosis, and myocardial infarction (Dikalova et al., 2020; Luo et al., 2022; Fan et al., 2023a). Some studies have found that SCU can protect vascular endothelial cells through different mechanisms and thereby exert cardiovascular protection (Table 3).

**TABLE 3:** Summary of experiments with SCU to protect the vascular endothelium.

Experimental model	Mechanism	Effect	Ref
MIR rats	PKG signal pathway	Enhances vascular endothelial relaxation and reduces myocardial infarction area	Li et al. (2015)
HR-induced injury of human cardiac microvascular endothelial cells	PKG signal pathway	Enhances endothelial cell viability and exerts vascular endothelial protective effects	Li et al. (2015)
HR-induced damage to human human cardiac microvascular endothelial cells	Promotes the expression of HSPD1, CCT6A and EIF6	Enhances endothelial cell viability and exerts vascular endothelial protective effects	Shi et al. (2015)
HR-induced endothelial dysfunction in rats	PKG signal pathway	Dilates coronary arteries vessels and repairs damage to the vascular endothelium	Chen et al. (2015)
High glucose-induced injury of HUVECs	PINK1/Parkin signal pathway	Enhances mitophagy, increases HUVEC cell vitality, and reduces vascular endothelial cell damage	Xi et al. (2021)
H_2_O_2_-induced damage to HUVECs	Reduces ROS and promotes the mRNA expression of SOD1 and Nox4	Reduces oxidative stress and exerts protective effect on vascular endothelium	Mo et al. (2018)
I/R-induced cardiac injury in mices	cGAS-STING signal pathway	Improves cardiac function and attenuates apoptosis	Li et al. (2023b)
H/R-induced damage to H9c2 cells	cGAS-STING signal pathway	Mitigates apoptosis	Li et al. (2023b)

CCT6A, chaperonin containing TCP1 subunit 6A isoform; EIF6, p27BBP protein; HSPD1, heat shock 60 kDa protein 1.

### 4.5 Anti-cardiac hypertrophy and fibrosis

Prolonged stress overload or noxious stimuli induce changes in the heart, such as cardiomyocyte hypertrophy and interstitial fibrosis, which macroscopically manifest as cardiac hypertrophy. Although cardiac hypertrophy is a physiological and pathological adaptive response, continued pathological stimulation can cause cardiac remodeling, leading to arrhythmias and heart failure (Marian et al., 2020; Fan et al., 2023a). Recent investigations have revealed that SCU has anti-myocardial hypertrophic and fibrotic properties (Table 4).

**TABLE 4:** Experimental summary of SCU against cardiac hypertrophy and fibrosis.

Experimental model	Mechanism	Effect	Ref
phenylephrine-induced hypertrophy in H9c2 and AC16 cardiomyocytes	Reduces TRAF2, NF-κB, p65, inhibits TRAF2, IκBα phosphorylation	Inhibits cardiomyocyte hypertrophy and resists cardiac hypertrophy	Shi et al. (2022)
Cardiac hypertrophy induced by PE or aortic banding	CaMKII signal pathway	Inhibits cardiomyocyte hypertrophy and resists cardiac hypertrophy	Pan et al. (2010)
MI rats	Inhibits FN1 increase and TGF-β1 expression	Reduces interstitial fibrosis and improves impaired cardiac function in infarcted rats	Pan et al. (2011)
AngII-induced proliferation of CFs	Inhibits the upregulation of FN1 and TGF-β1 and the phosphorylation of p38 MAPK and ERK1/2	Inhibits CF proliferation and collagen production, resists myocardial fibrosis	Pan et al. (2011)
ISO-induced myocardial fibrosis in rats	NOTCH signal pathway	Reduces myocardial fibrosis	Zhou et al. (2014)
DOX-induced chronic cardiotoxicity in rats	TGF-β1 signal pathway	Reduces myocardial fibrosis	Sun et al. (2023)

FN1, Fibronectin 1.

### 4.6 Regulation of glucose metabolism and lipid metabolism

Hyperglycaemia and hyperlipidemia are independent risk factors for CVD. The microvascular, macrovascular, and myocardial tissues of the human body will be harmed by long-term hyperglycemia, which will also hasten the development of cardiovascular disorders such as atherosclerosis, acute myocardial infarction, diabetic cardiomyopathy, and heart failure (Withaar et al., 2021; Paolisso et al., 2022; Rampin et al., 2022; Wei et al., 2022; Li et al., 2023a). Atherosclerosis is known to be facilitated by hyperlipidemia. However, it has been discovered recently that serum lipids can directly harm cardiac tissues by inducing oxidative stress, inflammatory reactions, and other processes that result in ventricular dysfunction and electrophysiological alterations (Castillo et al., 2018; Choi et al., 2021; Mohammadi-Shemirani et al., 2022). Therefore, reducing blood lipids and glucose levels is crucial to preventing the onset of cardiovascular illnesses. Numerous research conducted recently have supported the regulating effects of SCU on cholesterol and glucose metabolism (Table 5).

**TABLE 5:** Experiments on SCU regulation of glucose metabolism and lipid metabolism.

Experimental model	Mechanism	Effect	Ref
Db/db mices	Nrf2/HO-1 signal pathway	Increases HbA1c, insulin and pyruvate levels, improves glucose intolerance, and inhibits blood sugar elevation	Liu et al. (2019)
HFD/STZ-induced type 2 diabetes in mice	Inhibits FBG increase	lowers blood sugar	Huo et al. (2021)
HFD/STZ-induced type 2 diabetes in mice	-	Inhibits the increase of serum TC, TG and LDL and the decrease of serum HDL	Huo et al. (2021)
Non-alcoholic fatty liver disease rats	Promotes PI3K/AKT signal pathway, promotes Nrf2 nuclear translocation, HO-1 and NQO1 expression	Reduces TC, HDL-C and LDL-C levels	Fan et al. (2017)
HFD rats	-	Inhibits the increase in TC, TG and LDL-C levels, inhibits the decrease in HDL-C levels	Fu et al. (2019)
HDF male rats	Reduces SOD and NO, increases MDA	Reduces serum TC, TG and LDL-C, increases serum HDL-C	Mo et al. (2018)
Modeled adipogenesis *in vitro* in preadipocytes (3T3-L1)	Upregulates the expression of PPARα, downregulates the expression of PPARγ and C/EBPα	Reduces adipocyte differentiation and resists adipogenesis	Lu et al. (2013)

C/EBPα, The transcription factor CCAAT/enhancer binding protein α; EBPα, enhancer-binding protein alpha; HbA1c, Hemoglobin A1c; PPARα, Peroxisome proliferator-activated receptor α; PPARγ, Peroxisome proliferator-activated receptor gamma.

## 5 Improvement of bioavailability

Despite having a wide range of pharmacological actions and positive clinical therapeutic outcomes, SCU’s limited bioavailability still restricts its applications. Therefore, improving the bioavailability of SCU has become a hot research topic. A carrier substrate for a drug delivery system called a drug-encapsulated carrier is inserted into a matrix to create a tiny capsule that shields the active ingredient from the environment. The drug’s aqueous solubility, stability, and *in vivo* circulation half-life are all improved by this encapsulating technique. Some materials with good biodegradability, biocompatibility, and non-toxicity were selected as carriers for SCUs, such as nanoparticles, polymer micelles, liposomes, *etc.* (Table 6). The selection and application of these materials can improve the bioavailability of SCUs and further exert positive pharmacological effects.

**TABLE 6:** Parameters to improve SCU bioavailability.

Formulation	Carrier	Average diamete(nm)	Polymer dispersion index	Zeta potential	Entrapment efficiency (%)	Application	Ref
nanoparticle	PLGA	187.89 ± 3.42	0.077 ± 0.031	−6.99 ± 1.75 mV	63.63 ± 4.41	Anticerebral ischemia	Yang et al. (2022)
Liposome	S-UNL-E	156.67 ± 1.76	-	−28.77 ± 0.66 mv	-	Bone Builder	Minhua et al. (2022)
polymer	ε-PL-CD	200	-	8mv	-	Antitumor	Liao et al. (2020)
nanoparticle	Chitosan	200	0.5	25 mV	70	-	Liu and Ho (2017a)
nanoparticle	chitosan	182 ± 11	-	16.5 ± 3.1 mv	-	Antidiabetic	Wang et al. (2017a)
Liposome	CLN	181.0	-	23.8 mV	72.31 ± 1.96	Antiophthalmic disease	Wang et al. (2017b)
nanoparticles	bovine serum albumin	283.4	-	+17.95 mV	64.46	-	Wei et al. (2014)

PLGA, poly lactic-co-glycolic acid; S-UNL-E, scutellarin loaded on ultradeformable nanoliposome scutellarin EDTMP; ε-PL-CD, a novel b-cyclodextrin pendant polymer; CLN, characterize a cationic lipid nanoparticle.

Yang et al. (2022) found that SCU-loaded poly (lactic-hydroxyglycolic acid) (PLGA) nanoparticles (NPs) improved the bioavailability and therapeutic effect of SCU. Compared with free SCU, it prolongs the *in vitro* release spectrum and blood circulation duration of SCU, increases SCU levels in ischemic brain tissue, and significantly reduces cerebral infarction volume. In another study, nanoliposomal baicalin (S-UNL-E) was found to promote SCU-enabled modulation of bone metabolism, with high encapsulation rate and stability of S-UNL-E, as well as more effective promotion of osteogenic differentiation and bone formation compared to SCU(Lee et al., 2016; Minhua et al., 2022). In addition, it has been found that the encapsulated drug SCU:ε -PL-CD enhances the inhibition of tumor cell growth and tissue protection by SCU(Liao et al., 2020). Wang et al. (2021b) designed and synthesized a triglyceride-mimicking prodrug of SCU and demonstrated that it can effectively improve the bioavailability of SCU. By definition, prodrugs are derivatives or precursors of therapeutically active molecules. It can be biotransformed in the body through spontaneous processes, such as hydrolytic degradation or biocatalytic mechanisms, ultimately releasing active molecules and ultimately exerting medicinal effects (Zhou et al., 2022c). With the continuous development of molecular biology, active substances such as chitosan and cyclodextrins are also used as biocarriers to improve the therapeutic effect of SCU(Liu and Ho, 2017b; Liao et al., 2020). Administering SCU-encapsulated drugs at specific sites not only improves bioavailability but also provides better targeting of action. By delivering unique SCU-loaded HP-b-CD/chitosan nanoparticles (CD/CS-SCU-NPs) to the brain through the nose and mouth, LIU et al. boosted the amount of SCU accumulating there (Liu and Ho, 2017b).

## 6 Discussion

In clinical practice, it is easy to find some problems with conventional drugs for treating CVDs, such as a single therapeutic target that cannot intervene in the disease from a comprehensive perspective. There are some toxic side effects of certain drugs, such as gastrointestinal discomfort, loss of vision, headache, liver damage, renal damage, dry cough, angioedema, *etc.*, and even some drugs will increase the risk of developing cancer (Lin et al., 2020; Wilkerson and Winters, 2022). Nowadays, with the gradual increase in the understanding of the ingredients extracted from herbs and diets, the miraculous effects of these ingredients are increasingly being recognized.

SCU is the primary active substance in the flavonoid composition of Calendula officinalis, which has sound therapeutic effects on CVDs. SCU can intervene in cardiovascular system diseases through multiple signal pathways, including the TGF-β1/MAPK signal pathway, PI3K/AKT/mTOR signal pathway, Nrf2/Keap/ARE signal pathway, NOTCH signal pathway, *etc.* Among them, PI3K/AKT/mTOR, NOTCH, cGAS-STING, and CaMKII signal pathways have been the hot research pathways in the cardiovascular field in the last 5 years. SCU has the benefit of being a multi-target treatment and can protect various heart-related cell types, including cardiomyocytes, vascular endothelial cells, and fibroblasts. However, the study of multiple signal pathways in SCU is still in its infancy, and there are still problems, such as insufficiently comprehensive animal and human models, insufficiently in-depth study of pathway mechanisms, and insufficient clarity of signal relationships and interactions between pathways. Therefore, the types of disease models should be improved to expand the experimental scope and depth of research. SCU exerts protective effects against CVD by inhibiting inflammatory responses, alleviating oxidative stress, regulating apoptosis, protecting the vascular endothelium, resisting cardiac hypertrophy and fibrosis, and regulating glucose metabolism and lipid metabolism. However, some studies still need to be improved, such as the lack of relevant experiments to prove the exact mechanism of action of SCU on the regulation of lipid metabolism. Few studies have been done on the treatment and mechanism of action of SCU for cardiovascular system complications, including whether it can treat hypertensive renal damage, fundus changes brought on by hypertension, arrhythmia brought on by heart failure, heart failure coupled with hypoperfusion, *etc.* In addition, aging is an essential pathological factor that accelerates the development of cardiovascular disease, while population aging is a social problem shared by many countries around the world. Therefore, there is a need for research to explore the link between aging and heart disease to deal with heart disease aggravated or triggered as a result of aging. One study found that SCU can interact with SIRT6(Zhao et al., 2020). SIRT6, an important NAD-dependent enzyme, is vital in the regulation of both aging and heart disease (Guo et al., 2022; Nadeeshani et al., 2022). This suggests that SCU will have great potential for research and development in treating aging-related heart disease.

Recent studies have demonstrated the effectiveness of herbal compounds, including ginsenosides, curcumin, and cinnamaldehyde, in treating conditions like atherosclerosis, arrhythmia, and heart failure (Li et al., 2020; Luo et al., 2020; Sarhene et al., 2021; Lu et al., 2022). Research on these drugs is more comprehensive and in-depth, and studies on signal pathways and targets of action can be drawn upon to learn from further SCU studies. In addition, in clinical practice, it is often the case that the interactions of different herbal medicines are exploited to ingest multiple herbal ingredients at the same time. Different drug components may interact with each other to affect absorption efficiency. It has been found that the herbal constituents of Schisandra chinensis can promote the absorption and metabolism of ginsenoside, thus promoting the effects of ginsenoside (Liang et al., 2014). Additionally, Borneol can increase Geniposide’s bioavailability and targeting, while Rhein can increase Baicalin’s bioavailability by Inhibiting bcrp-mediated Baicalin Efflux Transport (Xu et al., 2014; Zhang et al., 2020c). Whether other drug components have an effect on the absorption and metabolism of SCU is likewise worth exploring and investigating.

Due to low bioavailability, the clinical application of SCU has been greatly limited. Although the development of drug encapsulation materials and carriers can effectively improve the bioavailability of SCU, there are still some problems, such as low drug loading capacity and poor targeting of the cardiovascular system. Therefore, developmental and experimental research in this area needs to be strengthened in the future. It is worth mentioning that a recent new study prepared poly (lactic-co-glycolic acid) nanoparticles (NPs) co-delivered with SCU and paeoniflorin (PAE) by an emulsification method. This method improved encapsulation efficiency and drug loading capacity, reduced nanoparticle size, better achieved therapeutic targets, improved cardiac function, and reduced cardiomyocyte apoptosis in rats (Yang et al., 2023). It is easy to draw some inspiration from this study. While focusing on the development of encapsulation materials, researchers can take advantage of drug-drug interactions to improve bioavailability and drug targeting.

In summary, SCU can modulate multiple signal pathways against heart disease and is a natural compound that combines antioxidant, anti-inflammatory, anti-apoptotic, and cardioprotective activities. Numerous experimental investigations have supported the effectiveness of SCU’s multi-targeted treatment of cardiovascular illnesses, indicating that its future application is promising. However, current research on SCU on CVDs has limitations, and the problems of low bioavailability need to be overcome. Based on the therapeutic efficacy, developmental potential, and research challenges of SCU, more systematic studies are needed to explore SCU to make them a cardiovascular drug with wide clinical application as early as possible.

## Figures and Tables

**Table:** 

**AKT**	protein kinase B
**ARE**	anti-oxidant response elements
**BAD**	bcl-2 agonist of cell death
**Bax**	BCL-2-associated X protein
**Bcl-2**	B cell lymphoma-2
**cGMP**	cyclic guanosine monophosphate
**CaMKII**	the multifunctional Ca^2+^ and calmodulin-dependent protein kinase II
**CVDs**	cardiovascular diseases
**CFs**	cardiac fibroblasts
**DCM**	diabetic cardiomyopathy
**DOX**	doxorubicin
**EBHM**	Erigeron breviscapus (Vant.) Hand-Mazz
**HO-1**	heme oxygenase-1
**HFD**	high fat diet
**HR**	hypoxia reoxygenation
**HUVECs**	human umbilical vein endothelial cells
**I/R**	ischemia-reperfusion
**ISO**	isoprenaline
**MAPK**	mitogen-activated protein kinases
**MIR**	myocardial ischemia-reperfusion
**mTOR**	mammalian target of rapamycin
**MyD88**	the adaptor protein myeloid differentiation primary response 88
**NF-κB**	nuclear factor kappa-B
**Nrf2**	nuclear factor erythroid 2-related factor 2
**Parkin**	parkin RBR E3 ubiquitin-protein ligase
**PINK1**	PTEN-induced kinase 1
**PI3K**	phosphatidylinositol 3-kinase
**p38**	the p38 group of MAP kinases
**SCU**	scutellarin
**SMAD**	the Smad transcription factors
**STAT**	signal transducer and activator of transcription
**STZ**	streptozotocin
**Tlr4**	toll-like receptor 4
**TGF-β**	transforming growth factor *ß*
**TNF-α**	tumour necrosis factor *a*
